# Supplementary material for: Evaluation of the Role of AID-Induced Mutagenesis in Resistance to B-Cell Receptor Pathway Inhibitors in Chronic Lymphocytic Leukemia
Source: Curr Issues Mol Biol. 2025 Dec 10;47(12):1031. doi: 10.3390/cimb47121031 (PMC12731823; doi:10.3390/cimb47121031)
Supplement: Supplementary file 1 [file cimb-47-01031-s001.zip › Pighi et al_supplementary figures legends_rev1.pdf]

Supplementary figures' legends:

Figure S1. **(A)** The graph shows AID-dependent mutation frequency in the control genes. Boxplots indicate cumulative frequencies of C>T or G>A transition mutation in DNA samples collected before (white) and after (gray) treatment in each patient (control n = 10, idelalisib n = 8, ibrutinib n = 11).

Figure S2 (A) Schematic representation of CRISPR/Cas9 targeting strategy for *AICDA* gene knock-out. In yellow exon 3 was targeted by sgRNA. The sgRNA target sequence and PAM domain are in blue and red, respectively. **(B)** Representative western blot analysis for AID protein in CRISPR/Cas9-derived MEC-1 cell lines. MEC-1 10_3 and 10_8 were selected as AID WT model, while 10_3.19 and 10_3.28 clones were selected as AID KO cell lines. β-actin was used as a loading control. In the present article MEC-1 10_6, 10_8, 10_3.19, and 10_3.28 clones are named as AID.WT1, AID.WT2, AID.KO1, and AID.KO2, respectively. **(C)** The graph shows sequencing analysis of the Cas9 cutting region in *AICDA* exon 3 of AID-KO MEC-1 clones. WES data confirms the complete deletion of a region of five base pairs leading to AID-KO. (D) Schematic representation of the experimental strategy employed to generate resistant clones from AID-WT (green) and AID-KO (red) cell lines by treating them with increasing concentrations of idelalisib (id) or ibrutinib (ib) for at least 6 months.

Figure S3. AID-WT and AID-KO MEC1 cells were cultured with idelalisib (orange/red) or ibrutinib (light blue/blue) with increasing doses up to 50 μM for 72 hours (for idelalisib: 50 – 25 – 10 – 5 – 1 – 0.5 – 0.1 μM; for ibrutinib: 50 – 25 – 15 – 10 – 5 – 1 - 0.5 - 0.1 μM). Cell viability was measured by Cell Titer-Glo® luminescent cell viability assay. Data was normalized to control (DMSO only). Mean values obtained from at least 3 independent replicates. Graphs and IC50 values were generated using GraphPad Prism 9.3 software.

Figure S4. **(A)** Cumulative mutation frequency of the analysed genes AID-WT (left) and KO (right) resistant MEC-1 cell lines. Boxplots indicate cumulative frequencies of C>T or G>A mutations in MEC-1 cells. **(B)** Mutation frequency of the analysed control genes in sensitive and resistant MEC-1 cell lines. Boxplots indicate cumulative frequencies of C>T or G>A mutations in MEC-1 cells (AID-WT and AID-KO). **(B)** Scatterplot from WES analysis showing Copy Number Variation (CNV) accumulated during the resistance acquisition in MEC-1 cell lines exposed to idelalisib treatment. In green are represented the positions with a decreased CNV and in red are the positions with a positive CNV. Only the chromosomes with altered CNV are shown in the figure.
