# Supplementary material for: Evaluation of the Role of AID-Induced Mutagenesis in Resistance to B-Cell Receptor Pathway Inhibitors in Chronic Lymphocytic Leukemia
Source: Curr Issues Mol Biol. 2025 Dec 10;47(12):1031. doi: 10.3390/cimb47121031 (PMC12731823; doi:10.3390/cimb47121031)
Supplement: Supplementary file 1 [file cimb-47-01031-s001.zip › Supplementary Figures_rev.pdf]

Figure S1.

A

AID off-target genes		control genes
c-MYC exon 1	LRMP	ICAM1
c-MYC exon 2	BCL7A	TFRC
RhoH/TTF	BACH2	LCP1
PAX5	SOCS1	MYBL1
PAX5-SE	IRF8	
PIM1	S1PR2	
BCL6	BIRC3	
TCL1A	CD74	
CXCR4	CD83	
IRF4	MS4A1 (CD20)	
BCL2		
MIR142		
BTG2		

B

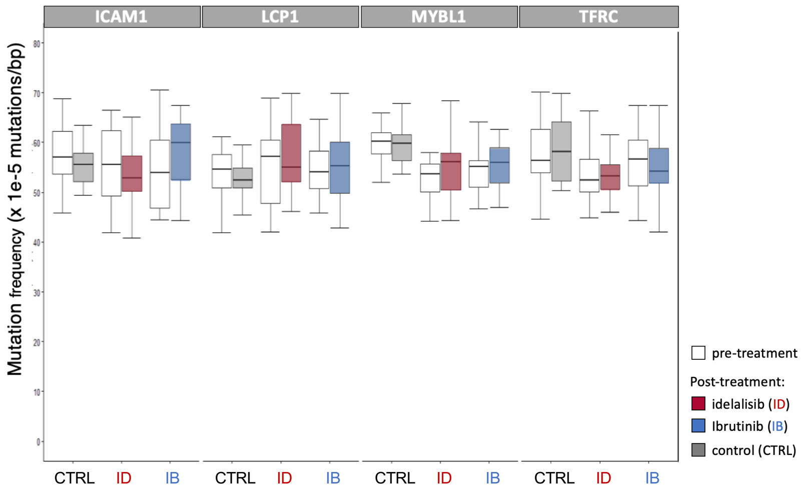

Figure S2.

A

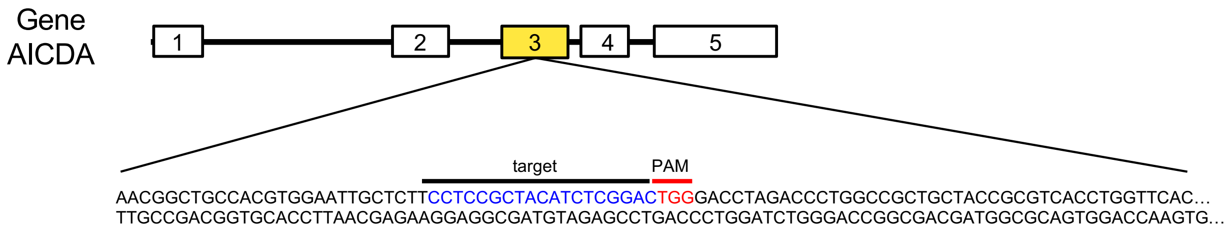

B

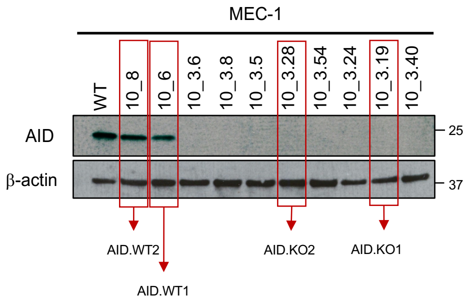

C

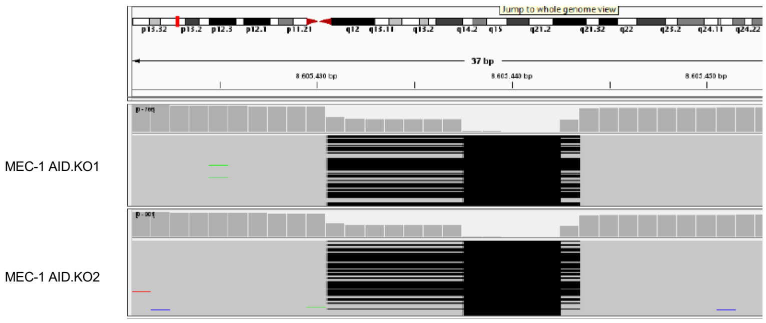

D

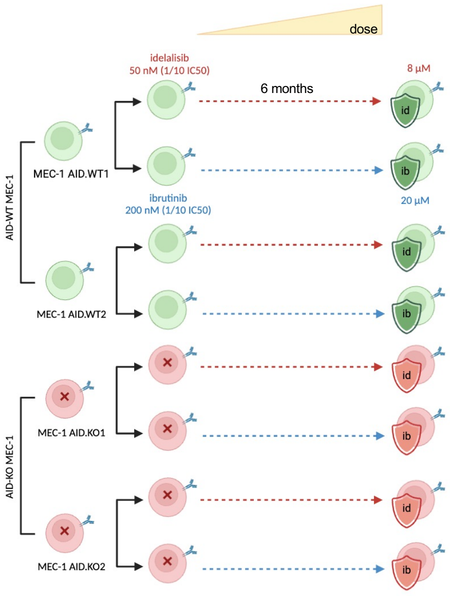

Figure S3

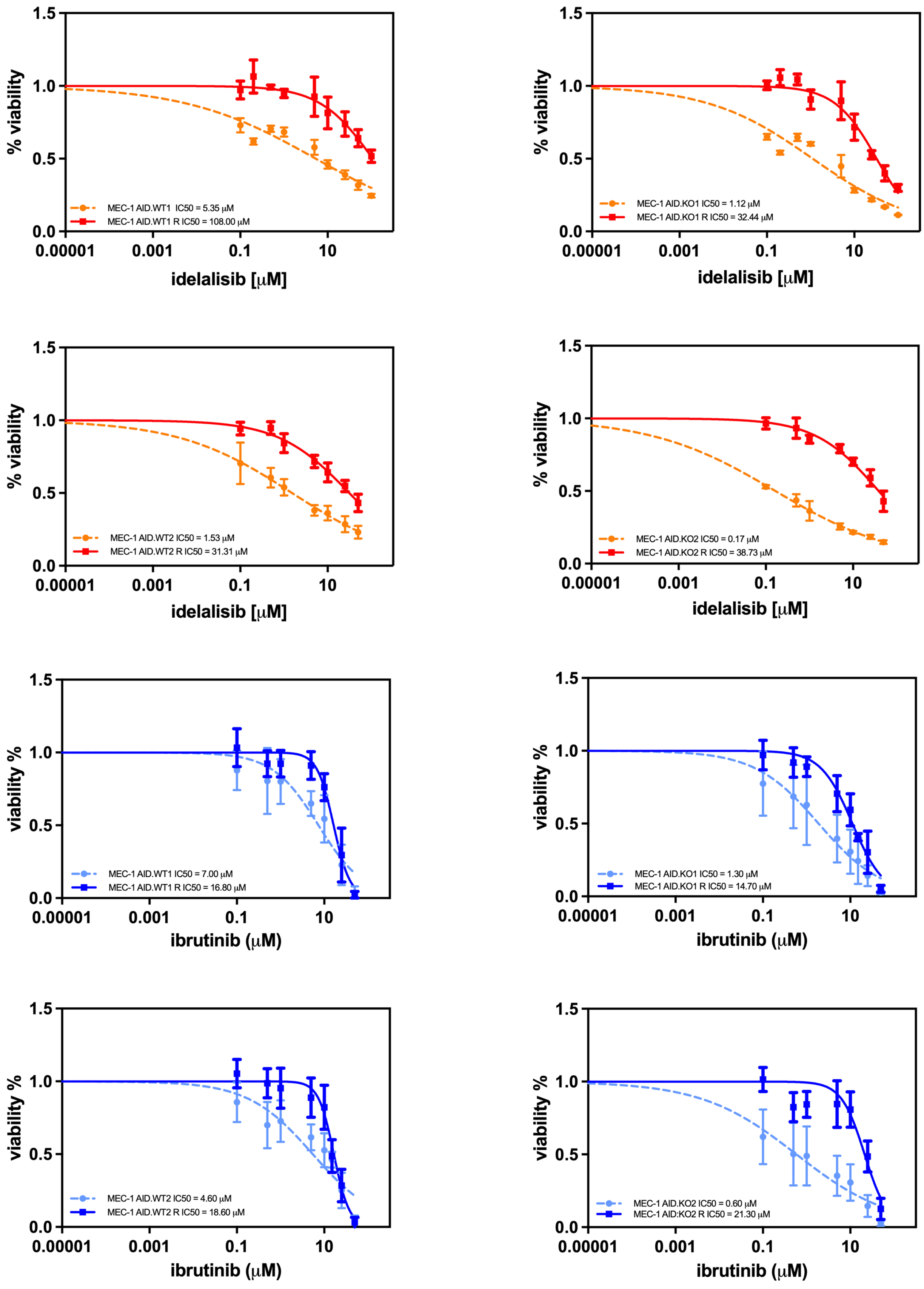
% viability
idelalisib [μM]
MEC-1 AID.WT1 IC50 = 5.35 μM
MEC-1 AID.WT1 R IC50 = 108.00 μM
MEC-1 AID.KO1 IC50 = 1.12 μM
MEC-1 AID.KO1 R IC50 = 32.44 μM
MEC-1 AID.WT2 IC50 = 1.53 μM
MEC-1 AID.WT2 R IC50 = 31.31 μM
MEC-1 AID.KO2 IC50 = 0.17 μM
MEC-1 AID.KO2 R IC50 = 38.73 μM
viability %
ibrutinib (μM)
MEC-1 AID.WT1 IC50 = 7.00 μM
MEC-1 AID.WT1 R IC50 = 16.80 μM
MEC-1 AID.KO1 IC50 = 1.30 μM
MEC-1 AID.KO1 R IC50 = 14.70 μM
MEC-1 AID.WT2 IC50 = 4.60 μM
MEC-1 AID.WT2 R IC50 = 18.60 μM
MEC-1 AID.KO2 IC50 = 0.60 μM
MEC-1 AID:KO2 R IC50 = 21.30 μM
0.00001
0.001
0.1
10
0.0
0.5
1.0
1.5

Figure S4.

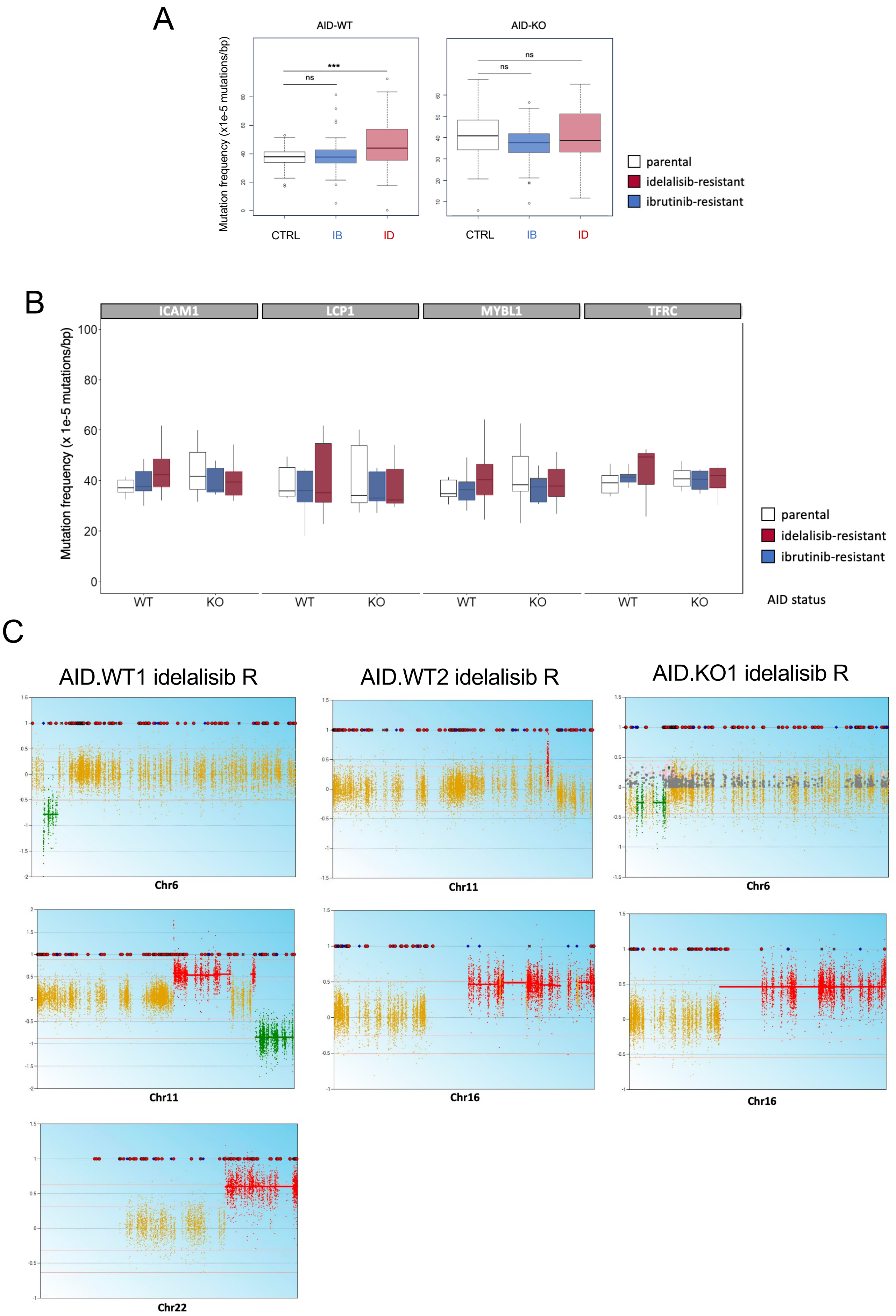
A
AID-WT
AID-KO
Mutation frequency (x1e-5 mutations/bp)
***
ns
CTRL
IB
ID
parental
idelalisib-resistant
ibrutinib-resistant
B
ICAM1
LCP1
MYBL1
TFRC
Mutation frequency (x 1e-5 mutations/bp)
100
80
60
40
20
0
WT
KO
AID status
C
AID.WT1 idelalisib R
AID.WT2 idelalisib R
AID.KO1 idelalisib R
Chr6
Chr11
Chr16
Chr22
