# Supplementary material for: Evaluation of the Role of AID-Induced Mutagenesis in Resistance to B-Cell Receptor Pathway Inhibitors in Chronic Lymphocytic Leukemia
Source: Curr Issues Mol Biol. 2025 Dec 10;47(12):1031. doi: 10.3390/cimb47121031 (PMC12731823; doi:10.3390/cimb47121031)
Supplement: Supplementary file 1 [file cimb-47-01031-s001.zip › Supplementary Table 1_rev.pdf]

Characteristic	CTRL	idelalisib	ibrutinib	Total population
n° patients	10	8	11	29
**Months 2 collection (mean ± SD)**				
n°	14.7 ± 6.16	11.37 ± 2.77	10.17 ± 3.13	12.46 ± 4.83
Unknown	0	0	5	5
**IGHV status**				
M	3	3	2	5
UM	3	3	9	12
Unknown	4	2	0	12
**TP53 status**				
M	2	3	4	8
WT	1	3	7	11
Unknown	7	2	0	10
**Progression**				
No	1	0	4	5
Yes	1	4	3	8
Richter +	1	1	0	2
Unknown	8	4	4	16

UM = not hypermutated; M = mutated/hypermutated; WT = wild-type
